# Exploring Bioactive Potential of *Streptomyces thinghirensis* WAE1 from Wadi El-Natron, Egypt

**DOI:** 10.1007/s12088-024-01215-8

**Published:** 2024-03-07

**Authors:** Mohamed E. Osman, Amany A. Abo-Elnasr, Eslam T. Mohamed

**Affiliations:** Department of Botany and Microbiology, Faculty of Science, Helwan University, Cairo, Egypt

**Keywords:** Actinobacteria, Antimicrobial, Antioxidant, Anti-inflammatory, L-asparaginase

## Abstract

This study aimed to investigate the bioactive metabolites produced by *Streptomyces thinghirensis* WAE1, an actinomycete isolated from El-Hamara Lake in Egypt. The discovery of new bioactive compounds from natural sources is crucial for the advancement of therapeutic treatments, and this study aimed to contribute to this field by exploring the potential of *Streptomyces thinghirensis* WAE1 as a source of such compounds. *Streptomyces thinghirensis* WAE1 was screened for its ability to produce antimicrobial, antioxidant, and anti-inflammatory metabolites. The results revealed that *S. thinghirensis* WAE1 exhibited strong antimicrobial activity against *Streptococcus pneumoniae* and moderate activity against *Listeria monocytogenes*, *Staphylococcus aureus*, and *Candida albicans*. *Streptomyces thinghirensis* WAE1 also displayed antioxidant activity through scavenging free radicals and chelating iron, and moderate anti-inflammatory activity as determined by its IC_50_ value. The isolate's demonstration of L-asparaginase activity suggests that *S. thinghirensis* WAE1 is a promising source of bioactive compounds with potential therapeutic uses. The high salinity and alkalinity of El-Hamara Lake, which create favorable conditions for the production of bioactive metabolites, further add to its potential as a source of actinomycetes strains with bioactive properties. These findings make both *S. thinghirensis* WAE1 and El-Hamara Lake valuable subjects for further exploration in the field of bioactive compounds.

## Introduction

The emergence of multi-drug resistance in microorganisms is a significant issue that calls for creating innovative and new antibiotics [1]. The existence of free radicals has been associated with chronic illness, inflammation, and cancer. These unstable molecules can cause chemical changes in biomolecules, leading to structural and functional modifications, when there is an overabundance of free radicals and insufficient antioxidant defense [2]. Natural compounds have indeed played a key role in the discovery of drugs for the treatment of many human diseases. The number of natural compounds discovered has surpassed one million, making it a vast and continuously expanding resource for developing new and effective treatments [3]. The genus *Streptomyces* is well-known for producing bioactive metabolites and is considered a key microorganism in the field of drug discovery. These microorganisms are filamentous, gram-positive bacteria with similarities in appearance to fungi. They have a large genome and a high G + C content, which allows them to adapt and survive in a range of environments [4]. Scientific research has shown that the genome of streptomycetes contains more than 20 clusters of synthetic genes involved in the biosynthesis of secondary metabolites. This genetic diversity is thought to be responsible for the production of varied bioactive metabolites. The wide range of metabolites produced by these microorganisms, including enzymes, antimicrobial, anticancer, and antioxidant agents, has led to a great deal of exploration and research for various applications, making streptomycetes important subjects for study in the field of drug discovery [5]. Researchers are still searching for new streptomyces species in understudied environments such as halophilic lakes in the hope of finding new compounds or therapeutic agents for disease treatment. These extreme environments have been shown to have the potential to produce novel antioxidant and anticancer compounds, emphasizing the importance of exploring these areas [6]. Additionally, L-asparaginase, an enzyme that plays a role in cancer therapy for acute lymphoblastic leukemia, works by reducing the supply of L-asparagine to cancer cells, thus hindering their growth and survival. However, there are challenges associated with its use, such as the occurrence of hypersensitivity reactions and the development of antibodies. To address these limitations, scientists have proposed alternative forms of L-asparaginase, specifically using Streptomyces species from the actinomycetes family as a source for its production [7]. Consequently, exploring streptomycetes from halophilic environments, like the Wadi El-Natron lakes in Egypt, holds great promise in uncovering new Streptomyces species and discovering valuable bioactive molecules.

## Material and Methods

### Isolation of *Streptomyces* Strain

The collection of *Streptomyces* spp. was carried out from soil samples sourced from various locations in Egypt through a dilution plate method using a medium of starch nitrate agar. After incubation at 30 °C for seven days, the most dominant actinomycetes isolates were identified, purified, and stored on slants of starch nitrate agar at a temperature of 4 °C for additional analysis [8].

### Antimicrobial Activity

A well diffusion assay was applied to investigate the antimicrobial effect of different bacteria (gram-positive and gram-negative) and fungi, including *Listeria monocytogenes* ATCC 19116, *Escherichia coli* ATCC 25922, *Clostridium sporogenes* ATCC 3584, *Staphylococcus aureus* ATCC 29213, *Salmonella enterica* ATCC 25566, *Cronobacter sakazakii* ATCC 29544, *Pseudomonas aeruginosa* ATCC 27853, *Klebsiella pneumoniae* ATCC 27736, and *Streptococcus pneumoniae* ATCC 49619 and *Candida albicans* ATCC 10231. The isolates were grown on starch nitrate agar for 7 days at 30 °C, and ten 5 mm agar plugs of each culture were inoculated into ISP2 broth medium in 1 L Erlenmeyer flasks, which were incubated for 14 days at 30 °C with shaking at 150 rpm. Cells were harvested by centrifugation at 5000 rpm and 4 °C. The supernatant was collected and extracted with ethyl acetate. The dried crude extract obtained after vacuum evaporation was dissolved in 90% ethanol to make a 50 mg/ml stock solution. Twenty microliters of each crude extract were transferred into a 5 mm well in nutrient agar plates seeded with the test organisms. The plates were incubated at 37 °C for one day for bacteria and 3 days for fungus and the inhibition zone around the holes was measured to determine antagonistic activity [9]. Due to the inhibitory activity observed, the crude extract was selected for further analysis of its biological activities.

## Identification of *Streptomyces* Isolate

### Morphological and Physiological Characteristics

After 14 days of incubation on starch nitrate agar at 30 °C, the strain WAE1's spore chain and surface were analyzed. The isolate was examined with a scanning electron microscope at various magnifications and evaluated for its biochemical and physiological properties using established methods [10].

### Genotypic Characterization

Using genomic DNA from the chosen strain, the 16S rRNA genes were amplified, and their sequence was established by Sanger sequencing. Through the BLASTn website, the final sequence was submitted to the gene bank database. Using MEGA version 4 software, the sequence was aligned, and the taxonomy categorization was established by building a phylogenetic tree [11].

### Gene Expression Analysis for PKS and NRPS

Degenerate primers were used in polymerase chain reaction analyses to detect polyketides (PKS) and non-ribosomal peptides (NRPS) genes in the actinomycete isolate. GelRed (Biotium) was used to visualize the PCR products on 1% agarose gel electrophoresis, and amplicon detecting was scored as positive if the amplicon was present and of the expected size (1200–1400 bp for PKS type I, 600–700 bp for PKS type II, and 700–800 bp for NRPS), or negative if the amplicon was absent [12].

## Biological Activity

### In-Vitro Antioxidant Activities

Crude extract at various concentrations (50, 100, 150, 200, and 250 µg/ml) antioxidants' ability to neutralize free radicals was measured using DPPH radical scavenging assay, ABTS radical scavenging assay, ferric-reducing antioxidant power (FRAP) assay, metal chelation assay, hydrogen peroxide radical scavenging assay, scavenging superoxide assay, nitric oxide radical scavenging assay and lipid peroxidation inhibition assay. 20 µg/ml, 40 µg/ml, 60 µg/ml, 80 µg/ml, and 100 µg/ml of ascorbic acid were used as the standard reference [13–18].

### Determining the Total Amount of Phenolic and Flavonoid Content

The WAE1 strain ethyl acetate extract's total phenolic content was calculated using the Folin-Ciocalteu method, and the aluminum trichloride method was used to determine the total flavonoid compounds. A 2 mg/mL DMSO solution of the crude extract was made, and gallic acid and rutin were employed as standards for the phenolics and flavonoids, respectively [19].

### In-Vitro Anti-Inflammatory Assay

RAW 264.7 murine macrophages were used in a scientific experiment to see whether an extract had anti-inflammatory properties. The cells were cultivated in a growth mixture containing fetal bovine serum and other supplements and then placed in microwell plates. The cells were exposed to the sample extract, a known anti-inflammatory compound (indomethacin), or an inflammatory inducer (LPS). After 24 h of incubation, the levels of nitric oxide (NO) produced by the cells were measured using the Griess assay. This assay involves converting nitrite, a stable byproduct of NO, into a colored compound that can be quantified by spectrophotometry at 540 nm. Using the Alamar Blue reduction test to measure cell viability, we analyzed the sample extract with the LPS-induced inflammation group and expressed the results as a percentage decrease in NO production. The experiment was performed in triplicate for each treatment group [20].

### Screening for L-Asparaginase Activity

A two-step screening procedure was used to assess the enzyme L-asparaginase production by WAE1 strain. The strain's ability to produce L-asparaginase was assessed qualitatively in the first phase using modified M9 agar plates, and quantitatively in the second. The quantity of ammonia generated in the reaction mixture, which was calibrated using standard ammonium chloride solutions, was used to measure the enzyme's activity. The enzyme’s activity was measured in units, where one unit equalled the quantity of enzyme necessary to produce 1 mol of ammonia per minute under specific conditions [21].

### Statistical Analysis

Three duplicates of each experiment were performed. The data's mean and standard deviation (SD) were used to express the findings by using excel software.

## Results and Discussion

### Isolation and Screening

Sixty actinomycete isolates were obtained from soil samples collected from various locations across Egypt. Following purification of these isolates in culture, their ability to produce antimicrobial metabolites was evaluated using an inhibition assay. Of the sixty isolates tested, nineteen (32%) demonstrated inhibitory activity against one or more pathogenic indicator microorganisms. The isolate producing the strongest antimicrobial effect, denoted WAE1, was selected for further characterization. WAE1 was isolated from a soil sample collected near El-Hamara Lake located in Wadi El-Natron, Egypt (30° 23′ 47.86′′ N, 30° 19′ 0.46′′ E; Fig. 6). To explore its biotechnological potential, WAE1 was grown under controlled conditions to induce the biosynthesis of secondary metabolites. Both qualitative and quantitative screening of extracts from WAE1 cultures revealed potent bioactivity, warranting further investigation into its biologically active compounds. Messaoudi et al. [22] isolated 40 strains of actinobacteria from saline environments and date palm rhizospheres in Algeria, including Streptomyces, Nocardiopsis, and Saccharopolyspora. The isolates showed moderate to strong antimicrobial activities against bacteria and fungi, indicating the potential of these rare actinobacteria for bioactive compounds. Yang et al. [23] isolated 77 actinobacterial strains from soil samples of the hypersaline lake Gudzhirganskoe in Siberia. Streptomyces dominated among the isolates, and 33% of them showed inhibitory activity against multidrug-resistant pathogens like *S. aureus* and *Acinetobacter baumannii*. This suggests that unconventional environment like hypersaline lakes, could be explored for discovering novel actinobacteria with potential therapeutic applications.

**Fig. 1:**
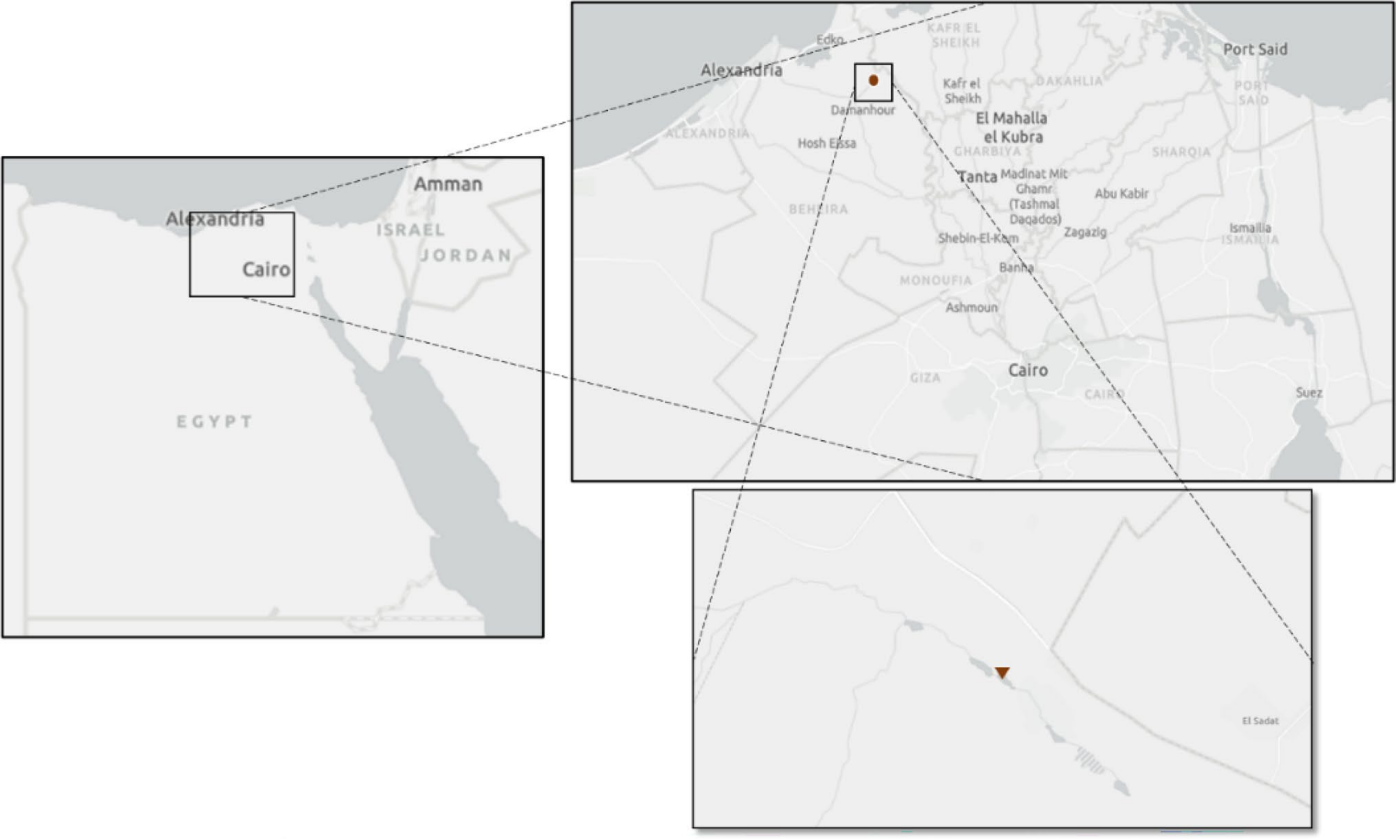
shows the location of El-Hamara Lake in Wadi El-Natron, Egypt, where the potent antimicrobial-producing actinomycete isolate WAE1 was obtained from a soil sample

### Bioactive Metabolites Activity

The WAE1 isolate demonstrated significant bioactive metabolite production, exhibiting antimicrobial activity against both bacterial and fungal test pathogens. Strong inhibition was observed against *S. pneumoniae* ATCC 49619 and *L. monocytogenes* ATCC 19116. Moderate activity was also seen against *S. aureus* ATCC 29213 and *K. pneumoniae* ATCC 27736, while weak activity was measured against *C. albicans* ATCC 10231. No inhibitory effect was found for *C. sporogenes* ATCC 3584, *S. enterica* ATCC 25566, *E. coli* ATCC 25922, *C. sakazakii* ATCC 29544, *P. aeruginosa* ATCC 27853. The antimicrobial metabolites produced by WAE1 showed differential activity against gram-positive and gram-negative bacterial strains as well as the fungus tested. The results are presented quantitatively in Table 1 and Fig. 1 to compare the antimicrobial potential of WAE1 against various pathogens. Al-Ansari et al. [24] studied the antibacterial activity of *Streptomyces* sp. AS11 against various pathogenic bacteria. The crude extract showed significant antibacterial activity, with zones of inhibition ranging from 10 to 29 mm. *S. aureus* was the most susceptible, with a zone of inhibition of 29 mm. Other pathogens like *Proteus mirabilis* and *Salmonella Typhi* showed zones of 20 ± 2 mm and 16 ± 2 mm, respectively. Jaroszewicz et al. [25] studied the antibacterial properties of *Streptomyces* strain M4_24 from the Szczelina Chochołowska cave. They found that strain showed antimicrobial activity against most tested bacteria, M4_24 produced inhibition zones ranging from 3.0 to 12.0 mm against *S. aureus*, *S enterica*, *Enterococcus*, *E. coli*, and *P. aeruginosa*, indicating its ability to secrete bioactive compounds.

**Table 1:** Antimicrobial activity of WAE1 isolate against pathogenic strains as measured by inhibition zone diameters (mean ± standard deviation, n = 3) in agar well diffusion assays

Microorganisms	Inhibition zone (mm)
*S. pneumoniae* ATCC 49619	33.17 ± 0.88
*L. monocytogenes* ATCC 19116	28.67 ± 1.15
*C. sporogenes* ATCC 3584	–
*S. aureus* ATCC 29213	12.33 ± 0.80
*K. pneumoniae* ATCC 27736	5.88 ± 1.04
*S. enterica* ATCC 25566	–
*E. coli* ATCC 25922	–
*C. sakazakii* ATCC 29544	–
*P. aeruginosa* ATCC 27853	–
*C. albicans* ATCC 10231	12.63 ± 1.13

–, no inhibition zone

**Fig. 2:**
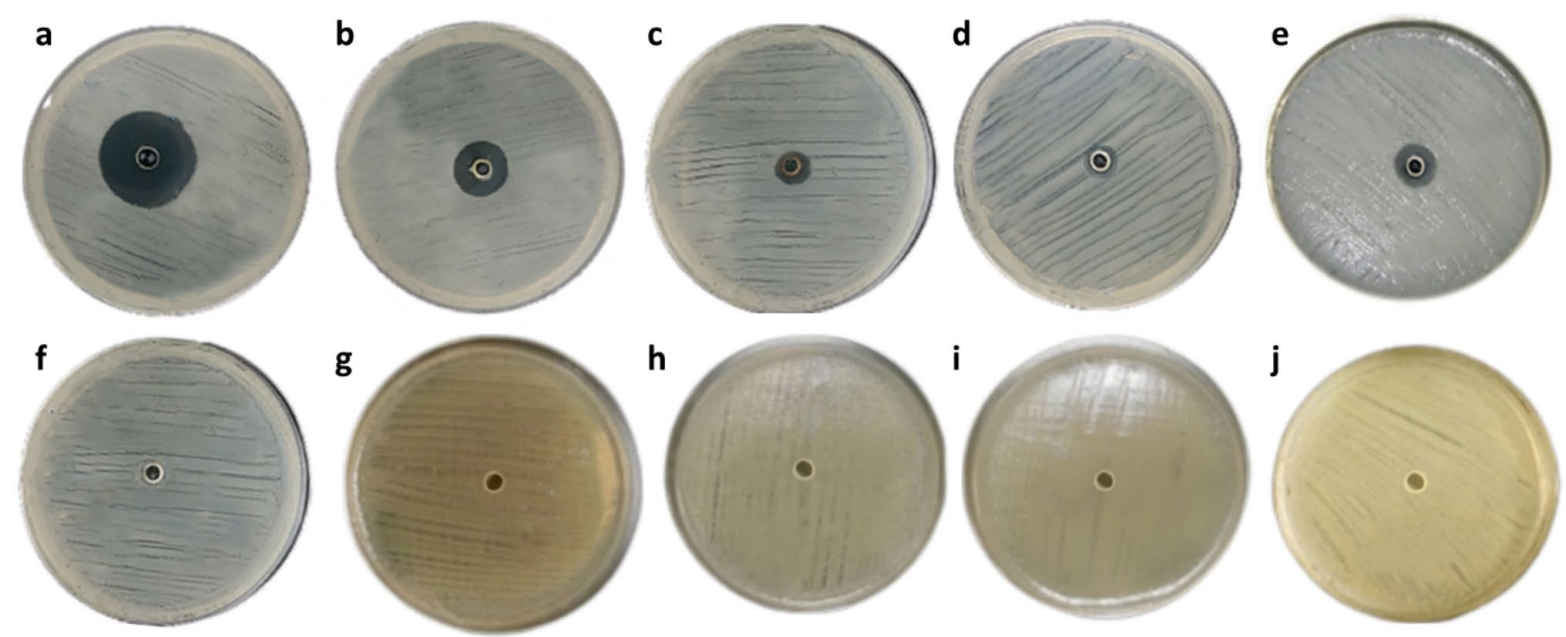
Antimicrobial activity of WAE1 isolate against various pathogenic bacterial and fungal strains as determined by agar well diffusion assays. The activities were evaluated against, **a** *S. pneumoniae* ATCC 49619, **b** *L. monocytogenes* ATCC 19116, **c** *S. aureus* ATCC 29213, **d** *K. pneumoniae* ATCC 27736, **e** *C. albicans* ATCC 10231, with no activity observed against, **f** *C. sporogenes* ATCC 3584, **g** *S. enterica* ATCC 25566, **h** *E. coli* ATCC 25922, **i** *C. sakazakii* ATCC 29544 and **j** *P. aeruginosa* ATCC 27853

### Identification of WAE1 Isolate

**Fig. 3:**
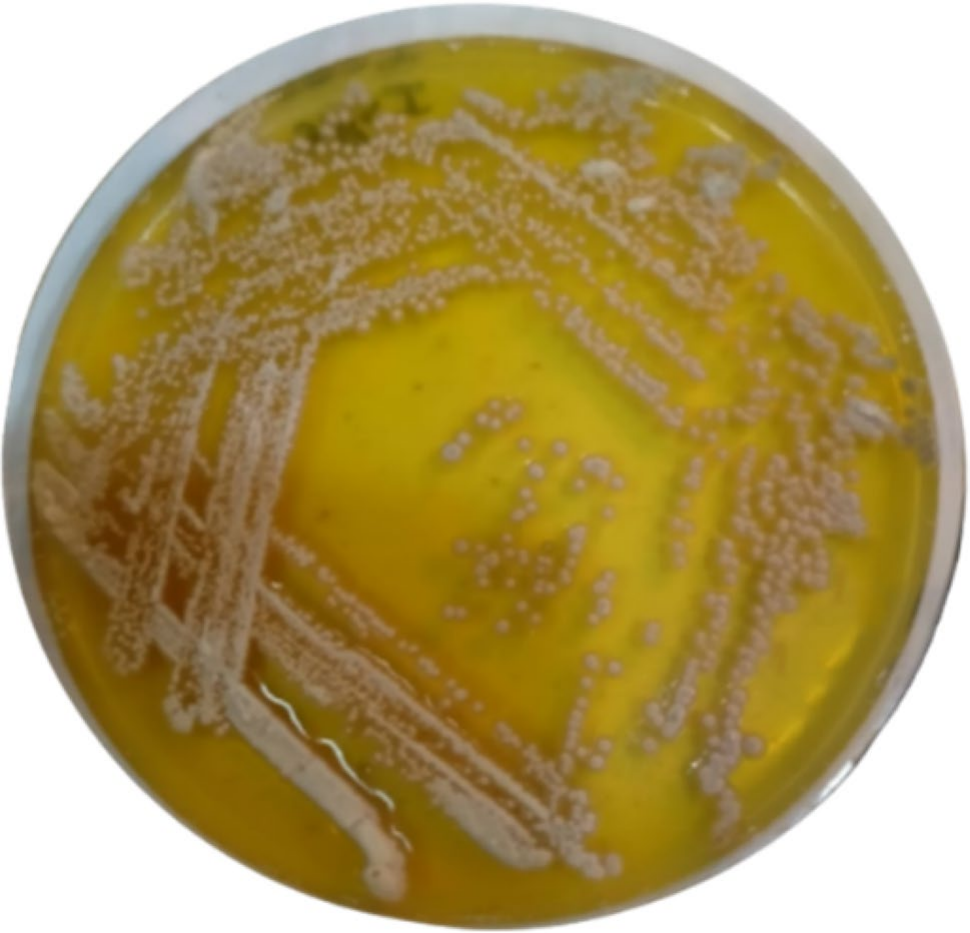
Phenotypic appearance of the actinobacterial isolate *S. thinghirensis* WAE1 grown on starch nitrate agar showing whitish-grey colonies exhibiting yellow-colored exopigment production

**Fig. 4:**
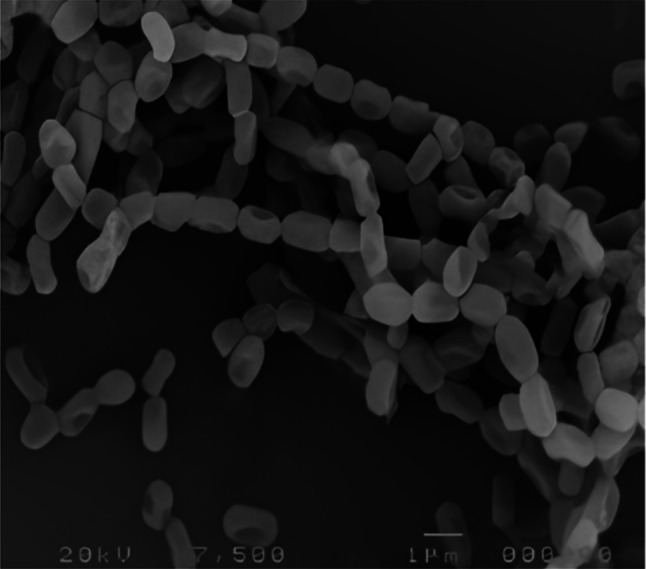
Phylogenetic tree of 16S rRNA gene sequence of WAE1 isolate and closely related type strains constructed using the neighbour-joining method, showing *S. thinghirensis* (NR_116901.1) as the closest type strain with 100% sequence similarity

Based on morphological, and biochemical characteristics and a polyphasic approach, indicate that the isolate is a member of the actinobacteria genus with novel characteristics (as shown in Figs. 2, 3 and Table 2). WAE1 strain was found to be closely related to *Streptomyces thinghirensis*, based on genomic identification (Fig. 4) which displayed typical morphological, coloration, and biochemical characteristics features of the *Streptomyces* genus and submitted into the GenBank database under the accession number ON584359.1 [26]. Almalki [27] isolated an actinomycete producer of polyketide antibiotics, SCA-7, from a marine sediment sample in Al-Khobar, Saudi Arabia. The isolate showed promising antimicrobial activity against *Enterococcus* sp. and was characterized for identification. Morphological analysis revealed filamentous growth with aerial mycelium, and biochemical characterization showed it could utilize various sugars and grow in different nitrogen sources and temperatures. Molecular identification confirmed SCA-7 belonged to the *Streptomyces* genus, exhibiting 99% sequence similarity to *Streptomyces felleus*. Maleki et al. [28] identified 12 *Streptomyces* isolates from soil samples in northwest Iran. Morphological analysis revealed high antibacterial activity by these isolates. Biochemical tests and 16S rDNA gene sequencing identified carbon source utilization patterns. Molecular identification showed 99% sequence similarity to *Streptomyces* species for all 12 isolates. RAPD-PCR clustering identified two promising isolates as *S. albogriseolus* and *S. coelicolor*. These findings confirmed the identification of *Streptomyces* species with potent antibacterial activity.

**Table 2:** *Streptomyces thinghirensis* WAE1's physiological and biochemical characteristics

Test		Result	Test		Result
Gram stain		+	Nitrogen source	potassium nitrate	+
Media	ISP 1	Whitish grey		Tyrosine	+
	ISP 2	Light grey		Cysteine	+
	ISP 3	Light grey		Tryptophane	+
	ISP 4	Grey		Arginine	+
	ISP 5	Whitish Grey	pH	5	–
	ISP 6	Light Grey		7	+
	ISP 7	Light Grey		9	+
Carbon source	Arabinose	+		11	–
	D-Xylose	+	Temperature (^o^C)	20	+
	Sucrose	+		30	+
	D-Mannitol	+		40	+
	Galactose	–		50	–
	Lactose	+	NaCl (%)	0	+
	Ribose	+		2.5	+
	Rhamnose	+		5	+
	Starch	+		7.5	+
	D-Glucose	+		10	–
	Fructose	+			
	Mannose	+

**Fig. 5:**
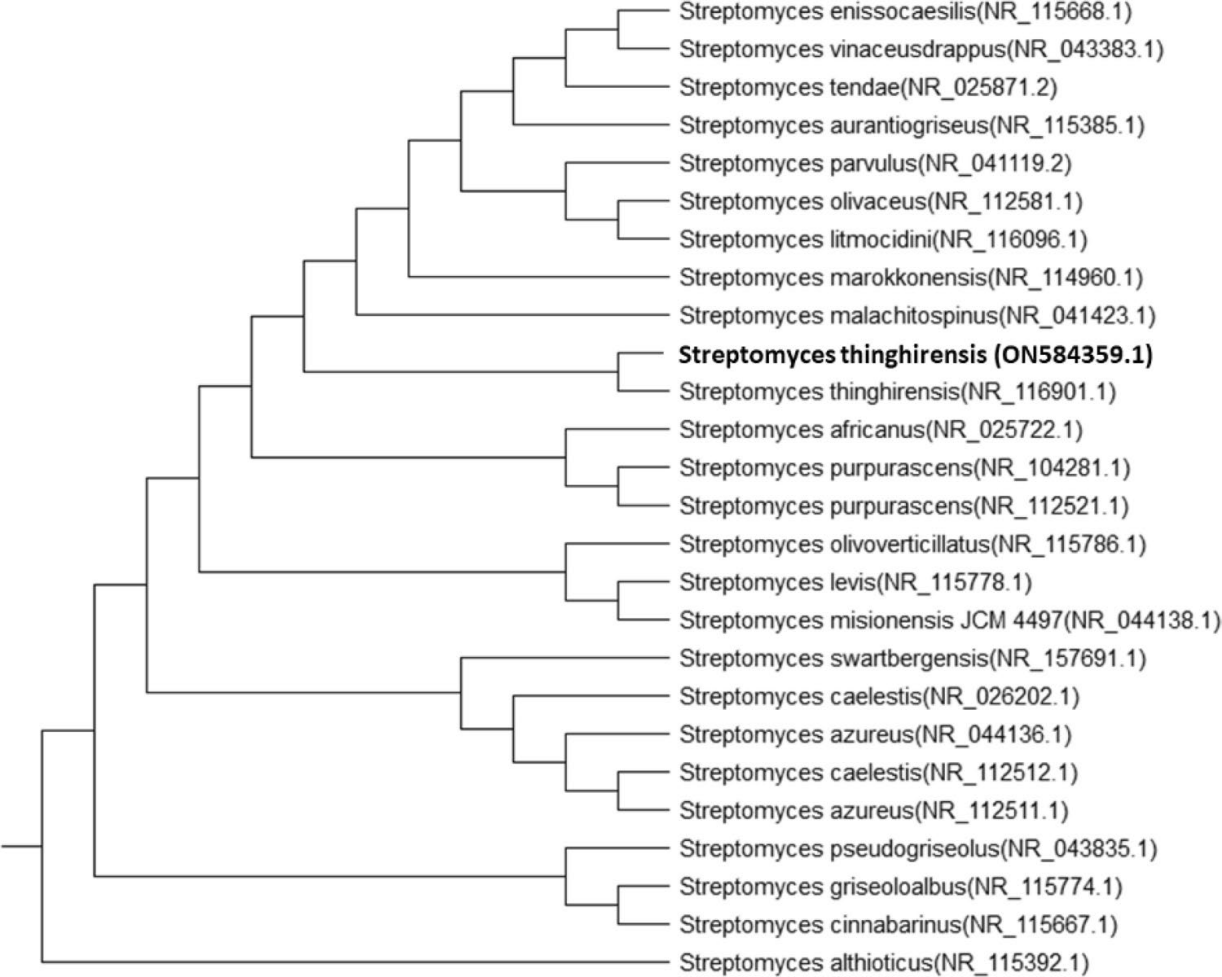
Phylogenetic tree of *S. thinghirensis* WAE1 was created by neighbour-joining technique

### Detection of PKS and NRPS Genes

The biosynthetic potential of the WAE1 isolate was assessed by screening for the gene sequences of non-ribosomal peptide synthetases (NRPS), polyketide synthases type I (PKS-I), and type II (PKS-II). The chromosomal DNA of the isolate was amplified using degenerate primers. This actinobacteria isolate was found to only include the adenylation domain of NRPS genes, as indicated by an amplicon size of 700–800 bp (Fig. 5). Non-ribosomal peptides genes have been documented to play a role in producing biologically active compounds such as antibiotics and antifungal metabolites, further strengthening the potential of *S. thinghirensis* WAE1 as a promising candidate for the discovery of new drugs [29]. Peng et al. [30] screened 42 *Streptomyces* isolates from rhizospheric soil of Panax notoginseng for biosynthetic genes encoding type I polyketide synthase (PKS I), type II polyketide synthase (PKS II), and non-ribosomal peptide synthetases (NRPS) using PCR with degenerate primers. They found that 41 of the 42 isolates yielded sequences related to at least one of the three gene types screened for. Specifically, they detected PKS I loci in 30 isolates, PKS II loci in 38 isolates, and NRPS loci in 7 isolates. Ghashghaei et al. [12] screened 22 actinobacterial isolates for genes involved in polyketide and non-ribosomal peptide biosynthesis. They found one isolate was positive only for the adenylation domain of NRPS genes, indicating the isolate's potential to produce secondary metabolites synthesized via NRPS gene clusters. This highlights the potential of these isolates in polyketide and peptide biosynthesis.

**Fig. 6:**
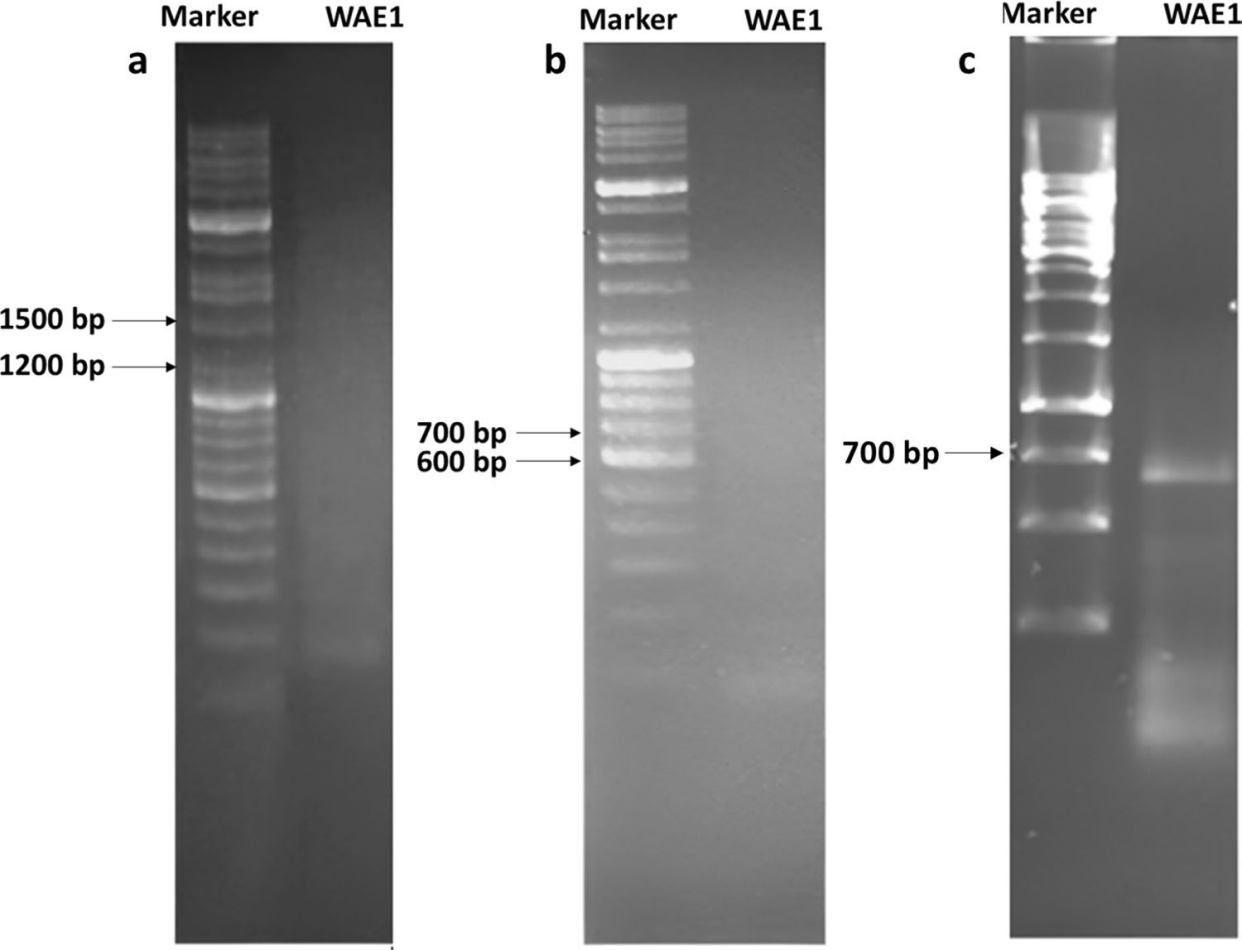
PCR amplification of PKS-I (**a**), PKS-II (**b**) and NRPS (**c**) gene sequences with an amplicon size of 1200–1400, 600–700 and 700–800 bp using primers K1F/M6R, KSαF/KSαR and A3F/A3R, respectively, on agarose gel 1%. The *S. thinghirensis* WAE1 isolate was found to only include the adenylation domain of NRPS genes, as indicated by an amplicon size of 700–800 bp

## Biological Activity

### In-Vitro Antioxidant Activities

**Fig. 7:**
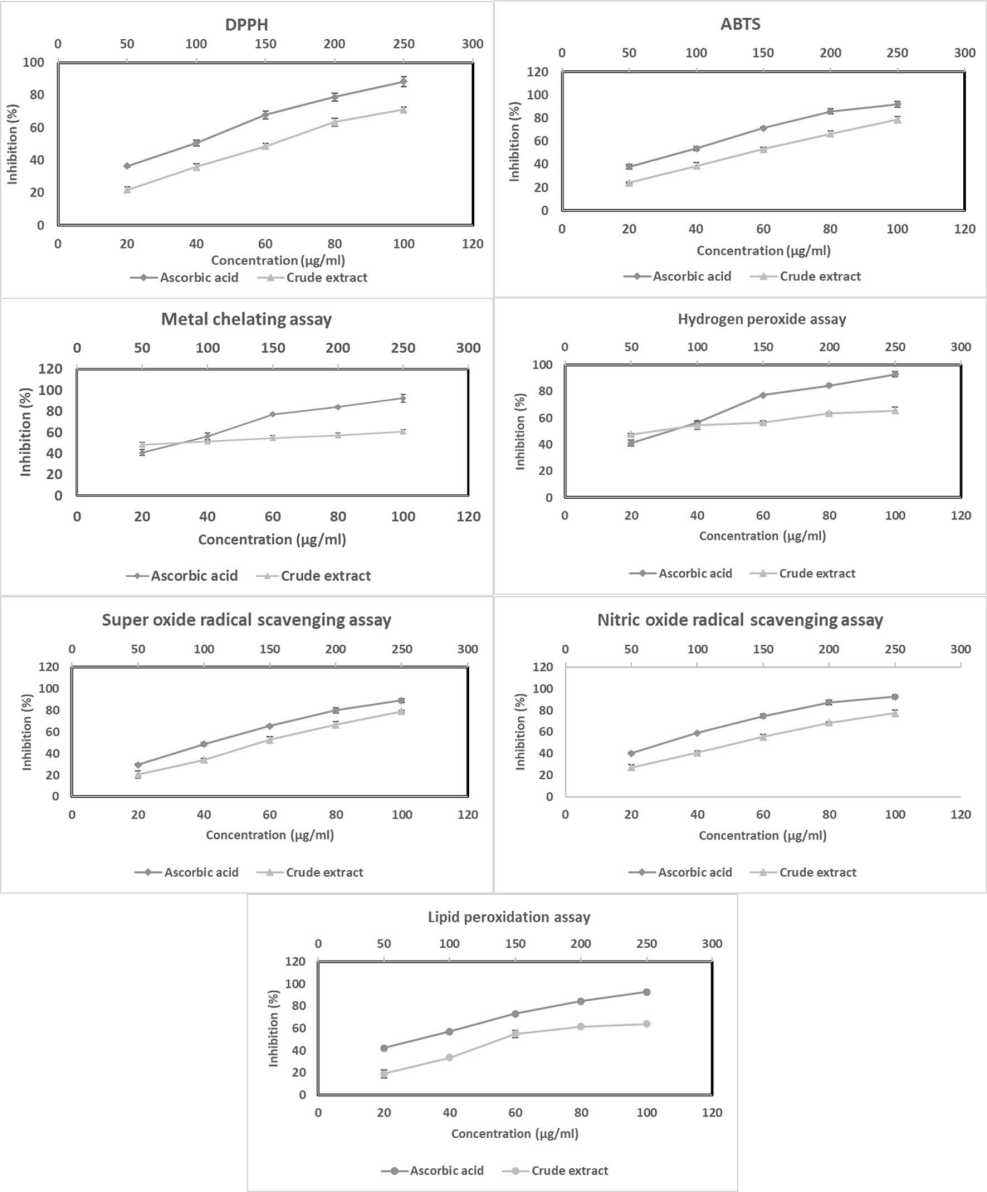
Assessment of the antioxidant properties of the WAE1 extract and ascorbic acid used as a control by using different antioxidant assays

The results demonstrated that, the WAE1 extract exhibited significant dose-dependent inhibition of DPPH, ABTS, and hydrogen peroxide radical activity, with IC_50_ values of 157.08 ± 2.66 μg/ml, 141.39 ± 3.46 μg/ml, and 66.01 ± 1.57 μg/ml, respectively. It also demonstrated metal chelating activity with an IC_50_ value of 76.58 ± 3.52 μg/ml and ferric-reducing antioxidant power with a value of 0.30 ± 0.02 mM Fe^2+^/g. The extract showed superoxide and nitric oxide radical scavenging activity with IC_50_ values of 148.29 ± 3.15 μg/ml and 135.14 ± 2.81 μg/ml, respectively. Additionally, it exhibited lipid peroxidation inhibition with an IC_50_ value of 164.38 ± 2.18 μg/ml. For comparison, the IC_50_ values for vitamin C were 38.06 ± 0.67 μg/ml, 33.94 ± 0.89 μg/ml, 29.10 ± 2.67 μg/ml, 29.32 ± 1.64 μg/ml, 1.615 ± 0.7 mM Fe^2+^/g, 43.43 ± 2.09 μg/ml, 28.87 ± 1.67 μg/ml, and 28.98 ± 1.26 μg/ml respectively. The results are shown in (Fig. 7). These antioxidant mechanisms help counteract reactive oxygen species (ROS) that can be pathologically generated. Overproduction of ROS has been correlated with elevated risk for certain diseases associated with oxidative stress. Therefore, natural extracts from actinobacteria exhibiting antioxidant properties may offer protective effects by neutralizing ROS [31, 32].

The extract obtained from the actinobacterium strain WAE1 was found to contain phenolic and flavonoid components that likely contribute to its antioxidant properties. The total flavonoid content of the WAE1 extract was quantified as 11.23 ± 3.15 mg rutin equivalents per 100 mg of extract, while the total phenolic content was determined to be 57.89 ± 2.67 mg gallic acid equivalents per 100 mg of extract. These results indicate that the WAE1 extract is rich in phenolic and flavonoid compounds. Previous studies have demonstrated a positive correlation between the total phenolic and flavonoid contents of microbial extracts with their antioxidant abilities, likely due to the ability of these compounds to scavenge free radicals [19].

Kemung et al. [33] studied the antioxidant potential of *Streptomyces* sp. MUSC 11 from mangrove soil in Malaysia. They evaluated the methanolic extract's antioxidant activity using various in-vitro assays. The results showed that the extract showed concentration-dependent radical scavenging activity, with maximum activities of 31.42 ± 1.00% and 7.27 ± 4.73% at 4 mg/mL. It also showed metal chelating activity of 21.61 ± 1.71% and ferric reducing equivalents of 3.001–3.521 ng ascorbic acid in the FRAP assay. Rammali et al. [19] studied the antioxidant properties of *Streptomyces* species isolated from cold soil sites in Morocco. They found that the E23-4 strain extract had the highest total phenolic content and flavonoid content. The antioxidant activities of the extracts were evaluated using DPPH and ABTS radical scavenging assays. All extracts showed lower DPPH radical scavenging activity compared to ascorbic acid, with no significant differences between extracts. In the ABTS assay, E23-4 had the highest activity at 35.79% inhibition. A significant positive correlation was found between total phenolic and flavonoid contents and antioxidant capacity, indicating that polyphenols significantly contribute to antioxidant properties. The study concluded that Streptomyces isolated from cold soil sites in Morocco produced metabolites with notable antioxidant activities, suggesting potential applications as natural antioxidants. Tangjitjaroenkun et al. [34] conducted an in-vitro study on the antioxidant properties of the ethyl acetate extract from *Streptomyces achromogenes* TCH4. They found that the extract had a total phenolic and flavonoid content of 107.20 ± 2.57 mg GAE/g and 44.91 ± 0.84 mg QE/g of dry extract, respectively. Phenolic and flavonoid compounds are known to contribute significantly to the antioxidant activities. The extract also demonstrated free radical scavenging activity using the DPPH and ABTS assays. In the DPPH radical scavenging assay, the extract showed concentration-dependent scavenging activity with an IC50 value of 299.64 ± 6.83 μg/mL, compared to the positive control BHT of 32.95 ± 0.26 μg/mL. In the ABTS radical scavenging assay, the extract inhibited the ABTS radical in a concentration-dependent manner with an IC_50_ value of 65.53 ± 0.95 μg/mL. The ferric-reducing antioxidant power (FRAP) of the extract was 822.76 ± 9.12 mM FeSO_4_.7H_2_O/g dry extract, indicating its potential in reducing oxidized species. Shobha et al. [35] studied the in-vitro antioxidant properties of a metabolite extracted from *Streptomyces* species KSRO-04. They assessed various antioxidant activities, including total phenolic content, flavonoids content, DPPH radical scavenging activity, metal chelating activity, total reductive capability, lipid peroxidation inhibition, superoxide anion scavenging, and nitric oxide scavenging. The metabolite showed moderate antioxidant activity in the DPPH radical scavenging assay, with an inhibition percentage of 14.39% at 125 μg/ml concentration. It also showed good metal chelating activity, inhibiting the formation of the ferrous-ferrozine complex with an inhibition percentage of 39.55% at 200 μg/ml. It also showed high nitric oxide scavenging activity of 79.25% at 125 μg/ml. Saravana et al. [36] evaluated the antioxidant properties of *Streptomyces lavendulae* strain SCA5 through in-vitro assays. The ethyl acetate extract (EA-SCA5) showed dose-dependent antioxidant activity in all assays, with IC50 values of 507.61 ± 0.66 μg/ml for DPPH radicals, 617.84 ± 0.57 μg/ml for hydroxyl radicals, 730.92 ± 0.81 μg/ml for nitric oxide, and 864.71 ± 1.15 μg/ml for superoxide anion radicals. This demonstrated *S. lavendulae* strain SCA5's ability to scavenge various free radicals and its potential as a natural antioxidant.

### In-Vitro Anti-Inflammatory Assay

**Fig. 8:**
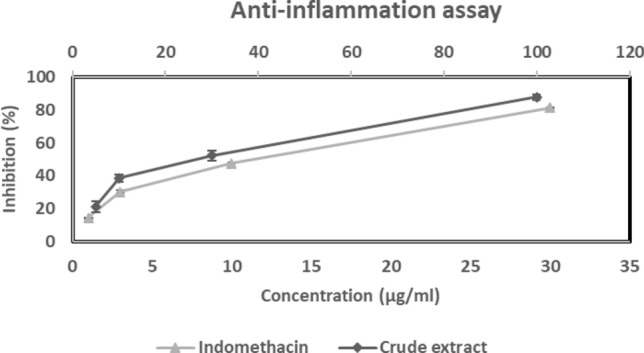
Anti-inflammatory activity of WAE1 extract and indomethacin used as a control

The LPS-induced NO generation by macrophages (RAW 264.7 cells) was significantly inhibited by WAE1 extract, with an IC_50_ value of 36.43 μg/ml. The IC_50_ value for indomethacin used as a control was 14.50 ± 0.75 μg/ml. The results are shown in (Fig. 8). This indicates that the strain has moderate anti-inflammatory activity. This is consistent with previous reports of anti-inflammatory activity from marine *Streptomyces* species isolated from the southeast coast of India [37]. Actinomycetes have been widely reported as a source of anti-inflammatory compounds in various studies [38, 39]. Xu et al. [40] found that a crude extract from mangrove sediment, *Streptomyces* sp. XY-R31, has significant anti-inflammatory effects. They used LPS-stimulated RAW264.7 macrophages as an in-vitro inflammation model and found that the R31 crude extract significantly inhibited LPS-induced nitric oxide (NO) production in a dose-dependent manner.

### Screening for L-Asparaginase Activity

**Fig. 9:**
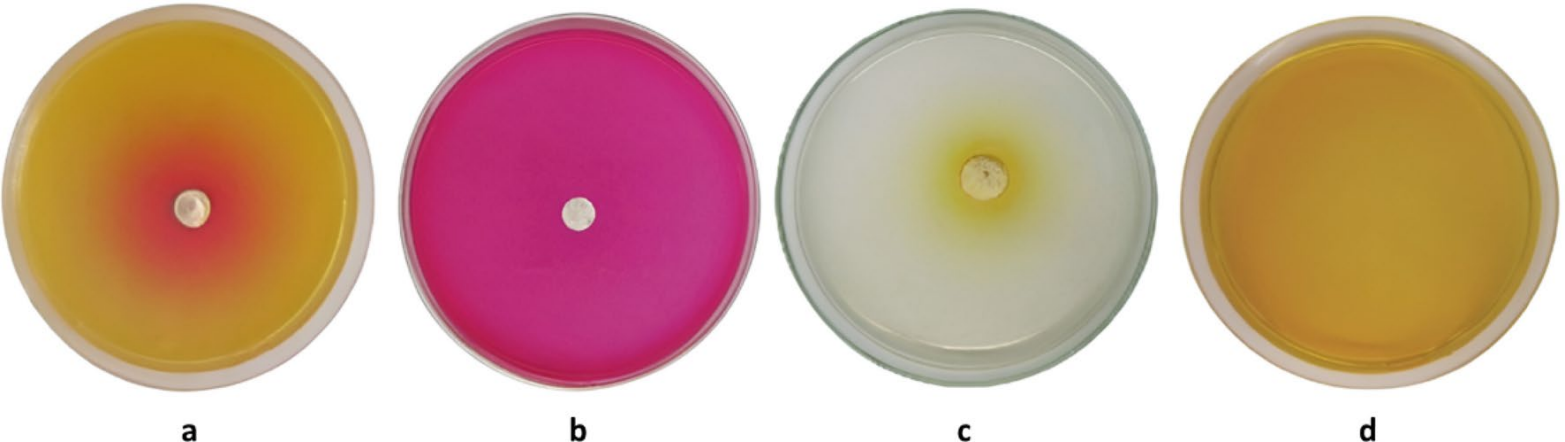
Qualitative detection of extracellular L-asparaginase secretion by *S. thinghirensis* WAE1 cultured in modified M9 solid medium. L-asparaginase activity was visualized using a pH indicator dye phenol red, where enzymatic hydrolysis of L-asparagine resulted in localized alkalinization and consequent color change from yellow to pink surrounding colonies after (**a**) 3 days and (**b**) 7 days of incubation. Control plates showed (**c**) inoculated medium lacking phenol red (positive control), and (**d**) uniform yellow coloration for uninoculated medium containing phenol red (negative control)

In a plate test, the L-asparaginase activity of WAE1 was determined by observing a change in color from yellow to pink around the colony (as seen in Fig. 9). L-asparaginase activity at 6 U/ml was detected in the WAE1 strain. This aligns with previous research that has shown that various actinobacteria can produce L-asparaginase, with *Streptomyces* sp. being the most frequently identified actinobacteria that can produce this enzyme from various sources. L-asparaginases have attracted a lot of interest because they may be used to treat acute lymphoblastic leukemia and in the food industry to prevent acrylamide production in fried and baked foods [41, 42]. El-Sabbagh et al. [43] discovered that a strain of *Streptomyces halstedii* from Egypt produces L-asparaginase. The production starts after 72 h of cultivation and reaches a maximum level of 3.9 U/ml after 120 h. Arévalo-Tristancho et al. [44] conducted a study on 78 actinobacterial strains from the Arauca riverbank in Colombia, focusing on their ability to produce L-asparaginase. They found that strain 112, identified as *Streptomyces lacticiproducens*, showed an L-asparaginase activity of 57.45 U/mL, indicating its potential as an effective producer.

## Conclusion

This study isolated and characterized an actinobacteria strain, WAE1, from a hypersaline lake in Egypt. A total of sixty distinct actinomycete isolates were obtained from soil samples, of which thirty-two percent showed antimicrobial activity in preliminary screening. Isolate WAE1 was selected for further characterization based on its superior antibacterial activity against pathogenic microorganisms. Morphological, biochemical, and molecular identification characterized WAE1 as *S. thinghirensis* strain. WAE1 demonstrated significant bioactive metabolite activities, particularly against *S. pneumoniae* ATCC 49619. Extracts from WAE1 also exhibited notable antioxidant activities in several in-vitro assays, indicating their potential as natural antioxidants. The isolate was found to harbor NRPS genes for secondary metabolite production and displayed moderate anti-inflammatory activity against LPS-induced NO generation in macrophages. Additionally, WAE1 produced the enzyme L-asparaginase, which has therapeutic applications. Overall, the results indicate that *S. thinghirensis* WAE1 is a promising candidate for the discovery of novel bioactive compounds with various biological activities. Further investigations are warranted to purify and characterize the active metabolites produced by this isolate.

## Data Availability

As corresponding author, I state that all data included in the manuscript are available.
